# Taxonomic and Functional Diversity of Leaves and Stem Endophytes of Eight *Agave* Species

**DOI:** 10.3390/microorganisms14020476

**Published:** 2026-02-15

**Authors:** Natalia Ysabel Labrín-Sotomayor, Patricia Alejandra Becerra-Lucio, Hugo Ruiz-González, Yuri Jorge Peña-Ramírez

**Affiliations:** 1 Sustainability Sciences Department, El Colegio de la Frontera Sur Unidad Campeche, Campeche 24500, Campeche, Mexico; nlabrin@ecosur.mx (N.Y.L.-S.); patricia.becerra@posgrado.ecosur.mx (P.A.B.-L.); 2 Freelance Consultant, Domicilio Conocido, Sisal 97356, Yucatan, Mexico; hruizg86.nopalito@gmail.com

**Keywords:** shotgun metagenomics, drought-tolerant species, tequila and mezcal production

## Abstract

More than 63% of Mexico’s territory is classified as arid or semiarid, where plants belonging to the genus *Agave* have evolved. Adaptation to drylands resulted from biochemical, physiological, and anatomical properties shared with other crassulacean plants; however, microbial symbionts also play critical roles in plants’ growth, health, and drought tolerance. To explore endophytic communities in *Agave* plants, we used a shotgun metagenomic approach. The taxonomic and functional diversity of endophytes were studied in the leaves and stem organs of *Agave americana*, *A.angustifolia*, *A. fourcroydes*, *A. karwinskii*, *A. potatorum*, *A. tequilana*, *A. cupreata*, and *A. rodacantha*. The microbial community structure did not differ significantly among species, regardless of geographic origin or local environmental conditions, whereas significant differences were observed between organs. We found 4058 genera shared among organs, of which 957 genera are exclusive to the stem and 492 to the leaves. The community analysis of stems and leaves identified bacterial genera, including *Acinetobacter*, *Klebsiella*, *Escherichia*, *Corynebacterium*, and *Streptomyces*. Significant differences were also observed between organs in the functional annotations. The dominant functional categories were associated with cell signaling and protein metabolism in both organs.

## 1. Introduction

The *Agave* (Asparagaceae) is a monocot crassulacean genus, endemic to Mesoamerica, commonly known as agaves or “*magueyes*”, with approximately 200 species, of which 166 species are present in Mexico, and ca. 72% of species are endemic to this country; therefore, Mexico has the highest diversity on the continent [1,2]. Agave plants can thrive in arid and semiarid ecosystems; their wide distribution has been crucial to people’s daily lives historically. In this context, agaves are among Mexico’s most important natural resources at the social, agronomic, and economic levels. Agaves have been used to produce fiber [3], ancestral fermented beverages [4], distilled beverages [1], food [5], and bioethanol [6]; however, their most significant economic use is in the production of spirit beverages.

The adaptation of agaves to arid and semiarid ecosystems has been facilitated by coevolutionary processes, in which symbiotic interactions with endobionts actively support agave survival in drylands [7]. Culturable endophytic bacterial species isolated from *A. tequilana* exhibit growth-promoting functional capabilities, such as nitrogen fixation, indoleacetic acid production, phosphate solubilization capacity, or antagonism against the *Fusarium oxysporum* AC132 phytopathogen. Some of them belong to *Acinetobacter* sp., *A. baumanii*, *A. bereziniae*, *Cronobacter sakazakii*, *Enterobacter hormaechei*, *Bacillus* sp., *Klebsiella oxytoca*, *Pseudomonas* sp., *Enterococcus casseliflavus*, *Leuconostoc mesenteroides* subsp. *mesenteroides*, and *Gluconobacter oxydans* [8].

Previous research indicates that endophyte communities can be affected by biotic and abiotic factors. For example, as occurs in other plant species [9], agaves may select and carry endophytes into specific compartments, like *Bacillus* sp. in the seeds, which may protect agave seeds from storage, desiccation, maturation, and germination; this species can also alter the root architecture of agave seedlings and serve as a nutrient reserve in poor soils [10]. Coleman-Derr et al. [11] reported the prokaryotic endospheric community associated with *A. tequilana* and *A. salmiana* subsp. Crassispina and *A. deserti* were more influenced by plant compartments and less by the geographic distance of the host species. However, the endophyte fungal composition was strongly influenced by the biogeographic factor. They found that although prokaryotic and fungal root endophytic communities could be differentiated among *Agave* species, a broader group of taxa was shared across them, representing 40% of the community. Other authors reported that the endosphere bacterial community in *A. salmiana* and *A. tequilana* was affected by season, which may support their role in arid-adaptation tolerance in these plants [12].

Previous evidence suggests that endophytes in agaves play a key role in the plant’s coevolutionary interactions with its associated microbial community. This tight relationship becomes significant when the microbial endophytic component is examined for its biotechnological potential. Microbial endophytes detected in the endosphere of agave plants are often present in the fermenting consortia of many *Tequila*, *Mezcal*, *Sotol*, and other agave-derived spirit as well as in *Pulque* (an agave-fermented sap) [13] factories, suggesting that the cross-contamination, very often in artisanal factories, may lead to the establishment and proliferation of endophytes in the fermentation tanks [14]. This finding is consistent with recent evidence showing that most microbial species in fermenting consortia can be detected in agave stems, regardless of the factory’s geographic location [15]. The whole microbial community from the fermentation also resulted in a composition very similar to that found in the endosphere of *Scyphophorus acupunctatus* (Coleoptera: Curculionidae), the boring weevil whose larvae feed exclusively on the stem tissue of agave plants [16], which suggests a coevolutionary process involved in agavines (an agave-specific type of carbohydrate), sap, and other biopolymers degradation with this insect [17].

In this work, we conducted a detailed exploration of taxonomic and functional microbial endophyte diversity in the stems and leaves of eight Agave species, including species and stem samples that have not been studied before. To deepen our knowledge of the factors that can modulate the endophyte community, including their potential in environmental adaptation and in the spirit industry, we decided to use a shotgun sequencing approach to identify the presence and relative abundance of microorganisms, including (1) those taxa previously reported to participate in fermentation; (2) the taxonomic groups uncommonly reported as endophytes due to the limits of amplicon-based approaches like virus, archaeans, oomycetes, microalgae, and protozoans; (3) the genomic approach enables us to perform the functional annotation of genes, exploring the metabolic potential of the native microorganisms of *Agave* plants in physiological adaptation to drought conditions, as well as those potential metabolic routes relevant to aromatic molecules in spirits beverages as well as those involved in the presence of undesirable molecules, such as methanol, furfural, or long-chain alcohols.

## 2. Materials and Methods

In this study, we analyzed the genomic datasets previously reported by our group [15,18] derived from endophyte populations in the stems and leaves of eight *Agave* species (Table 1). Briefly, representative wild plants (*n* = 3) were collected in the Mexican state of Oaxaca (16°33′ N, −96°49′ W, 1450 masl), except for *A. cupreata* plants, which were collected in the Michoacan state (19°27′ N, −101°11′ W, 1725 masl). Plants were sampled during plant cropping for *Mezcal* production (mature 8- to 12-year-old plants), obtaining leaves (≈9 square cm from the central part of the leaf (*penca*, in local terminology) and 5 cm from the base) and stem (≈27 cubic cm from the stem [*piña* or *mezonte* in local terminology]) organs by triplicate from each individual. Starting from 10 g of surface-sterilized tissue (3 min wash with 3% commercial sodium hypochlorite, followed by 3 rinses with sterile water), we aseptically extracted the sap/juice fraction by squeezing the tissue in a sterilized domestic lemon squeezer. From the pooled “juice” from 3 plants and 3 replicate samples, 200 µL were used for eDNA extraction using the ZymoBIOMICS DNA Miniprep Kit (cat D4300, Zymo Research Co., Irvine, CA, USA). Once purified, eDNA was sequenced as an external service at Novogene Co., (www.novogene.com). We uploaded raw *.fastq files to the KBase platform [19] for bioinformatic analysis. To perform quality assessment and read trimming, we use Trimmomatic v0.39 [20]. For pairing library objects and assembling, we use metaSPADES v3.15.3 [21] and CONCOCT v1.1 [22] for binning. To assign the taxonomic identity, we used Kaiju v1.9.0 [23] and the NCBI database for prokaryotes and eukaryotes. Gene annotation was performed by EggNOG mapper v2.1.9 and the DIAMOND algorithm [24]. The ecological indices were calculated from the taxonomic abundance matrix using PAST v4.17 [25]. Using PAST, we performed a Permutational Multivariate Analysis of Variance (PERMANOVA) using the Bray–Curtis similarity index. The significance threshold for PERMANOVA was set at *p* < 0.05, and 9999 permutations were used. NMDS analysis and Principal Component Analysis (PCA) were performed using a variance-covariance matrix and 1000 bootstrap replicates. We also performed a classical clustering analysis using the unweighted pair-group method with arithmetic averages (UPGMA) based on the Bray–Curtis similarity index, with 9,999 bootstrap replicates. The Venn diagram was created in Venny 2.1.0. [26].

## 3. Results

### 3.1. Taxonomy Diversity of Endophytes Associated with Agave Species and Compartments

We analyzed endophytic communities in the stems and leaves of eight Agave species using deep genomic sequencing. We processed a total of 782 million reads (360 million from stems and 422 million from leaves), equivalent to 58.5 Gbp, with an average read length of 150 bp and a mean Phred quality score of 33 or higher.

**Figure 1:**
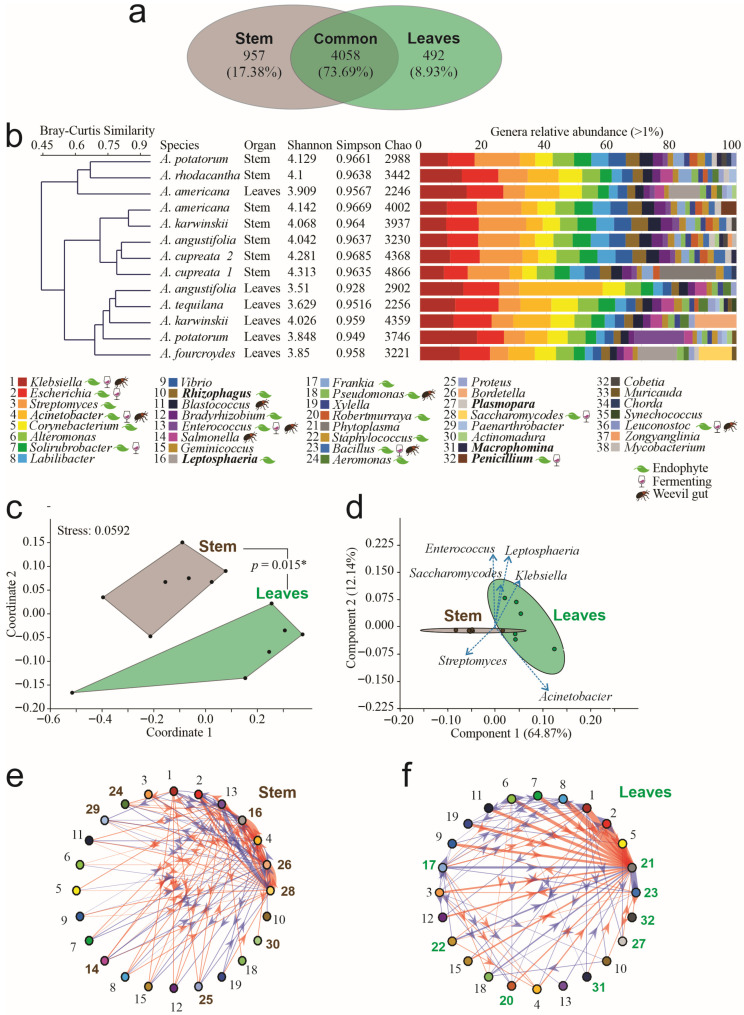
Taxonomic analysis of endophytes in agave leaves and stems. (**a**) Venn diagram of genera by tissue. (**b**) Bray–Curtis clustering of microbial communities, alpha diversity indices, and relative abundance of genera > 1% by sample. Legend names in plain text correspond to bacteria, names in bold correspond to fungi. Please refer to the references in Supplementary File S1 for previous reports of genera as endophytes, fermenters, or isolated from weevils’ gut. (**c**) Non-Metric Multidimensional Scaling (NMDS) analysis of genera by compartment. Connecting lines between polygons correspond to the *p*-value obtained by PERMANOVA analysis. (**d**) Principal Component Analysis (PCA) of genera by compartment. (**e**) Interaction networks in leaves. (**f**) Interaction network in stems. In networks, thickness corresponds to interaction strength, whereas color indicates red = positive interactions; blue = negative interactions, and the arrow indicates the interaction direction. Arial bold numbers correspond to genera that participate exclusively in each network (* = *p* < 0.05). Numbers in bold Arial correspond to the genera participating exclusively in each network.

The taxonomic assignment of the entire dataset yielded four domains, 27 kingdoms, 88 phyla, 233 classes, 523 orders, 1048 families, and 5507 genera. Stems and leaves shared 4058 genera, equivalent to 74% (Figure 1a). Exclusive genera were identified by organ, and 957 genera were exclusively from stems, among which *Entrophospora*, *Desemzia*, and *Candida* were the most abundant. In leaves, 492 exclusive genera were identified, among which Candidatus *Kryptobacter*, *Lentimonas*, and *Ekhidna* were the most abundant (Figure 1a). Thirty-nine genera had a relative abundance per sample greater than 1%, among which we found that the bacteria *Klebsiella*, *Escherichia*, *Streptomyces*, *Acinetobacter*, and *Corynebacterium* were the most abundant and common genera, widely dominating the microbiome; however, five fungal genera were present in a relative abundance > 1% per sample (Figure 1b). The Bray–Curtis clustering analysis detected three groups at 0.6 of similarity: the first cluster was composed of stem endophytes of *A. americana*, *A. karwinskii*, *A. angustifolia*, and *A. cupreata*; the second cluster grouped leaf endophytes of *A. angustifolia*, *A. americana*, *A. karwinskii*, *A. potatorum*, and *A. fourcroydes*; and the third cluster grouped leaves and stem endophytes of *A. americana*, *A. potatorum*, and *A. rhodacantha* (Figure 1b). The non-exclusive genera of endophytes were common among all leaf samples. Only seven genera were exclusive to and shared in all stem samples, forming a putative core of the *Agave* stem, comprising *Blepharisma*, *Cladocopium*, *Gordonibacter*, *Bailinhaonella*, *Paucilactobacillus*, *Loigolactobacillus*, and *Phlebia*; all of which had an abundance below 1% per sample. The first and second ones belong to the Chromista Kingdom, specifically to the Blepharismidae and Symbiodiniaceae families.

At the genus level, high levels of Alpha diversity, measured by the Shannon–Wiener (*H*′) index, were detected, ranging from 3.6 to 4.3. We found that the microbial communities between the species did not differ significantly (Duncan post hoc test, *p* = 0.3285). By kind of organ, Alpha diversity was higher in the stem samples than in leaves, averaging 4.26 and 3.92, respectively (Figure 1b), resulting in significant differences between the microbial communities present in those organs (Duncan post hoc test, *p* = 0.0012 **). The lowest diversity of endophytes was detected in the leaves of *A. angustifolia* (*H*′ = 3.51, Chao = 2902), and the highest diversity was found in the *A. cupreata* stem (*H*′ = 4.3, Chao = 4866) (Figure 1b). The Simpson index indicated dominance by a few genera, ranging from 0.928 to 0.9685 (Figure 1b).

The ordination NMDS analysis of the data showed two distinct polygons, grouping microbial communities by tissue, with a stress level of 0.059. Significant differences (*p* < 0.015 *) were detected by the PERMANOVA test (Figure 1c). The PCA showed a significant adjustment by components. The first two components explained 77% of the data diversity (Figure 1d). Using network analysis, we predicted microbial interactions and inferred greater interaction complexity in leaves than in stems. The leaves’ microbial communities exhibited 105 interactions, among which 57% were positive (Figure 1e), whereas the stem communities had 80 interactions, among which 58% were negative (Figure 1f). Moreover, we identified eight genera that exhibited significant exclusive participation in the network derived from the leaf samples. From these, the genera *Leptosphaeria*, *Bortedella*, and *Saccharomycoides* obtained the highest interaction forces. In the stem samples network, another eight genera with exclusive participation were identified; in this case, the genera *Phytoplasma* and *Bacillus* had the highest interaction forces.

**Figure 2:**
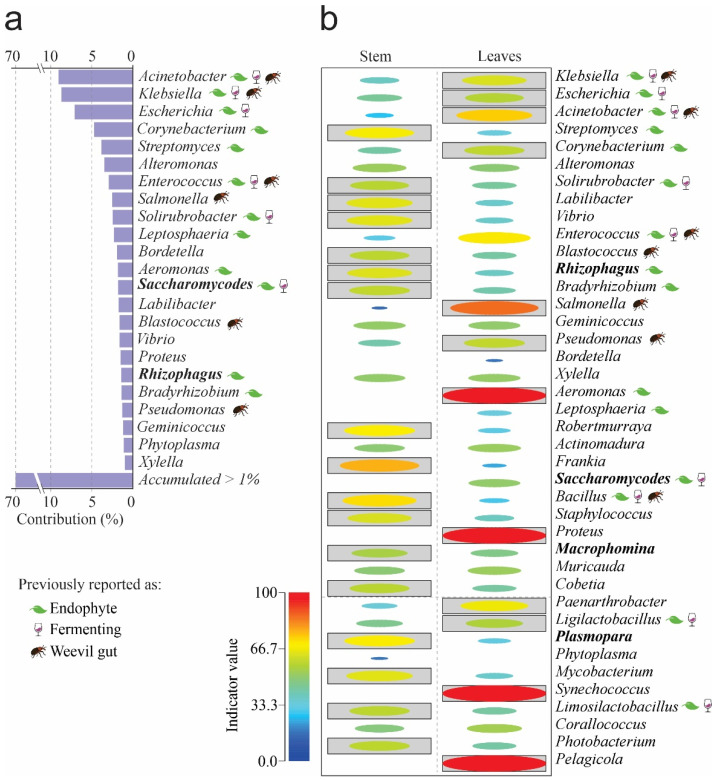
SIMPER and indicator taxa by organs at the Genus level. (**a**) SIMPER. (**b**) Indicator genera. Boxed ellipses correspond to significant differences = *p* < 0.05. The leaf, cup, and weevil icons represent previous role reported for any microbial genera. Please refer to Supplementary File S1 for references on previous reports of genera as endophytes, fermenters, or isolated from weevils’ gut.

To determine each species’ contribution to the observed dissimilarity, we used a similarity percentage analysis (SIMPER). Our results show that 23 genera accounted for 70% of the differences between samples, with *Acinetobacter*, *Klebsiella*, *Escherichia*, *Corynebacterium*, and *Streptomyces* making the most significant contributions, accounting for 35% (Figure 2a). The genera *Aeromonas*, *Proteus*, and *Shynechococcus* had the highest indicator values in leaves; the genera *Frankia*, *Streptomyces*, *Robertmurraya*, and *Bacillus* were the indicator species in stems (Figure 2b).

### 3.2. Functional Orthologous Categories of Endophytes Associated with Agave Species and Compartments

**Figure 3:**
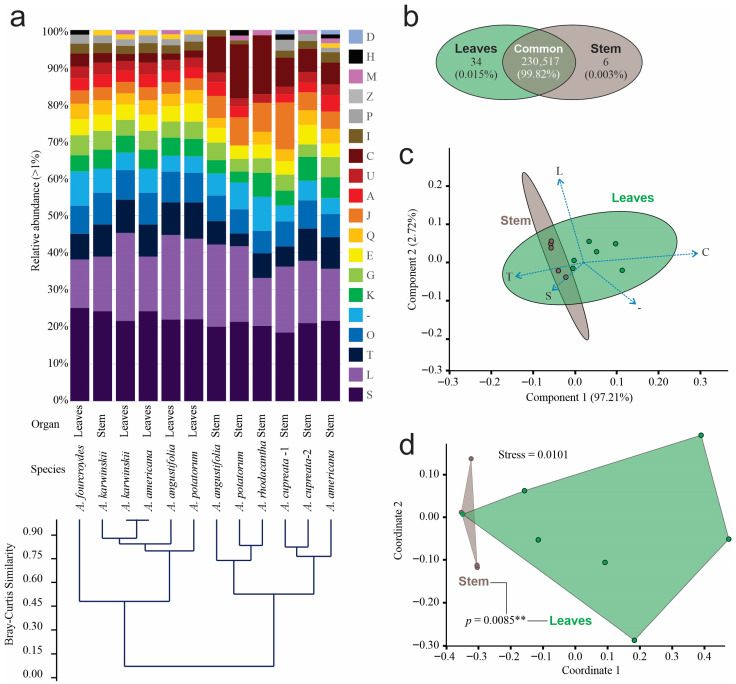
Relative abundance of functional orthologous groups of endophyte communities. (**a**) Relative abundance greater than 1% of relative abundance of functional categories of prokaryotic and eukaryotic grouped by Bray–Curtis clustering analysis: Functional orthologous categories are (A) RNA processing and modification, (C) energy production and conversion, (D) cell cycling control and mitosis, (E) amino acid metabolism and transport, (G) carbohydrate metabolism and transport, (H) coenzyme metabolism, (I) lipid metabolism, (J) translation, (K) transcription, (L) replication and repair, (M) Cell wall membrane and envelope biogenesis, (O) post-translational modification, protein turnover, chaperon functions, (P) inorganic ion transport and metabolism, (Q), secondary metabolites biosynthesis, transport, and catabolism, (S) function unknown, (T) signal transduction, (U) intracellular trafficking and secretion, (-) unclassified; (**b**) Venn diagram of functional orthologous categories by tissue; (**c**) PCA of functional orthologous categories; (**d**) NMDS analysis of functional orthologous categories. The connecting lines between polygons correspond to the *p*-value obtained from the PERMANOVA analysis (** = *p* < 0.01 ).

According to the Clustered Orthologous Gene (COG) classification, we identified 152 known functional subcategories in prokaryotic and eukaryotic organisms, among which 18 had relative abundances greater than 1% (Figure 3a). Replication and repair (L), signal transduction (T), and post-translational modification, protein turnover, and chaperone functions (O) were the most abundant. The Bray–Curtis clustering analysis grouped the data into two clusters at a similarity of 0.45. The first cluster consisted of leaf samples and the stem sample from *A. karwinskii*, while the second cluster grouped only stem samples. The annotated COG abundance in the leaves of *A. americana* and *A. karwinskii* was the most similar at the functional level (Figure 3a). Stems and leaves shared 99.8% functional COGs, 34 functions, equivalent to 0.015% were exclusive for leaves, and six COG functions, or 0.003%, were exclusive to stems (Figure 3b).

The first two components of the ordination analysis by PCA explained 100% of the data diversity as being signal transduction (T), as well as energy production and conversion (C), representing the functional orthologous category with higher contribution in the first component, and replication and repair (L), constituting the orthologous category with higher contribution in the second component (Figure 3c). SIMPER based on Bray–Curtis distances identified four COGs responsible for 54.4% of the differences between organs, where energy production and conversion (C) accounted for 21.27%; replication and repair (L) for 15.55%; translation (J) for 11.06%, and signal transduction (T) for 6.51%. The number of COGs in the leaf samples was higher than that of the stem samples in all cases, except in *A. karwinskii*, where the number of orthologous genes was slightly higher than that of the stem of the same species. The first two dimensions of the NMDS analysis also grouped functional orthologous genes by organ, with a stress level of 0.0101. The statistical analysis of orthologous genes revealed a significant difference (PERMANOVA, *p* = 0.0085 **) by organ (Figure 3d).

## 4. Discussion

In our research, we focused on the taxonomic and functional analysis of the endophytic community in leaves and stems from eight *Agave* species using a deep genomic analysis approach. We selected the *Agave* species based on their different levels of domestication, of which *A. tequilana*, with the highest level of domestication and lowest genetic diversity due to almost exclusive clonal propagation for extensive monospecific cropping [10]; *A. fourcroydes*, used as a fiber source, is also a domesticated species reproduced mainly by vegetative methods and scarcely by seeds, also being a commercial crop [10,27]. Low diversity has also been reported *in A. angustifolia* and *A. americana*, which are semidomesticated species used for distilled beverage production and as a fiber source; both are often propagated by seed or vegetative structures and are cropped in agroforestry systems [10,28]. The rest of the species considered in this work are used for *Pulque*, spirits, ornaments, fibers, food, and living fences; however, these species are mainly wild, tolerated, or transplanted from the wild. *A. karwinskii* and *A. potatorum* spontaneously self-reproduce through natural means, including both sexual and asexual strategies, whereas *A. cupreata* and *A. rhodacantha* only reproduce by sexual seeds [2]. Besides the heterogeneous degree of domestication and cropping intensity among the evaluated agaves, our results showed that the level of domestication, and therefore the cropping system, is not a significant factor in the diversity and structure of the endophyte community, at least for the more abundant ones. As previously reported [12], the endophytic microbiota appear to be represented by a consistent core of microorganisms, whose resilience may be a consequence of evolutionary selection mechanisms, allowing particular species to colonize each of these tissue-specific endospheres symbiotically. In contrast, when epiphytic or rhizospheric microbial populations have been evaluated, they exhibit greater variability according to cropping intensity and degree of plant domestication [11]. In our case, across the eight species in our scope, we did not detect any significant differences in endophyte alpha diversity. Moreover, geographically associated differences in endophytes were not detected between *A. cupreata* plants collected in the Michoacan state and those collected in the Oaxaca state, more than 500 km apart.

Looking for beneficial culturable *Agave* endophytes responsible for plant growth promotion and pathogen control, previous work has reported the isolation of 300 endophytic strains grouped in eight bacterial genera: *Acinetobacter*, *Cronobacter*, *Enterobacter*, *Bacillus*, *Klebsiella*, *Pseudomonas*, *Enterococcus*, *Leuconostoc*, and *Gluconobacter* [8]; all these genera were also identified in our samples; however, in our case, *Cronobacter*, *Enterobacter*, *Leuconostoc*, and *Gluconobacter* showed a relative abundance below 1%, which is congruent with previous results which demonstrated that some bacteria species can show a significative effect in the plant growth even when they are below 1% of relative abundance [29]. Our results detected dominant genera like *Klebsiella*, recently recognized in a meta-study as a phytohormone producer, nutrient solubilizer, and pathogen controller [30]; *Escherichia*, a genus that has also been previously reported as an endophyte from *Elaeis guineensis*, which appears to participate in plant nutrient uptake [31]; *Acinetobacter*, a widely recognized endophyte producer of plant growth regulators such as indole acetic acid, and gibberellins; as well as phosphate, potassium, and zinc solubilizer [32]. Other relevant bacterial genera identified in our work, such as *Streptomyces*, *Pseudomonas*, *Bacillus*, and *Staphylococcus*, have been reported as plant growth-promoting endophytes with key roles in drought tolerance [33,34]. The fungal genera *Rhizophagus* [35] and *Penicillium* [36], detected in the endosphere of the Agave’s stem and leaves, have also been reported as plant growth-promoting endophytes. Consistent reports of microorganisms found as plant endophytes, which play roles in plant growth and pathogen defense, reinforce the theory that plants actively modulate their endophytic populations. Consequently, this may explain the similarity in microbial populations across the evaluated *Agave* species, regardless of their geographic origin. Similar results evaluating agave endophytes were reported previously, where cultivation status did not affect microbial structure [12], whereas significant differences were found in endophyte communities from agave leaves and roots [11,12]. More recently, similar results were reported for *Agave utahensis*, where endophytes from roots and leaves showed distinct structures, confirming the existence of tissue-specific filtering of microbial taxa [37]. On the other hand, it has been reported that specific microbes co-occur in both the endosphere of agaves and the gut of the borer weevil *Scyphophorus acupunctatus*, the primary pest of agaves [38], as well as in the fermenting must for Mezcal production [15]. These co-occurring taxa, such as *Klebsiella*, *Acinetobacter*, *Enterococcus*, *Bacillus*, and *Leuconostoc*, appear to support the theory that agave endophytes may act as opportunistic saprotrophs in the degradation of complex polymers within *Agave* plants. Moreover, agave endophytes may be contributing by cross-contamination (possibly mediated by weevils, frequently attracted by the cooked juice in artisanal factories) to the spontaneously formed fermenting consortia, which in turn may represent an underappreciated source for bioprospecting microorganisms with interesting catalytic capabilities for the bioconversion of plant biomass to ethanol, or microorganisms involved in the production of the wide variety of secondary metabolites obtained during the spontaneous fermentation of cooked agave juices. On the other hand, ecological interactions between microbes, which regulate most metabolic and physiological processes, can be modeled by co-occurrence network analysis [39]. These networks may help unveil the relevant regulatory roles of less abundant microorganisms, as well as identify those acting in a different ecological guild under contrasting environmental conditions, or, as in our case, under different tissue-specific filtering endospheres. Our network analyses revealed that five key taxons, which participate only in leaf or stem networks, exhibited the highest interaction forces, including the negative interaction between *Bacillus* and *Phytoplasma* in the stem network. Previous reports have identified that *Bacillus mycoides* may help control the Phytoplasma responsible for Leaf Yellowing in *Vinca* plants [40], reinforcing the utility of these predictive models in generating potential biocontrol alternatives, as seen in this example. However, it is essential to complement network models with functional information to elucidate putative metabolic or physiological phenomena. Besides the scientific exploration to come, practical approaches can be explored by bioprospecting microorganisms from the agave endosphere. Culturable organisms showing in silico ecological antagonistic interactions could be tested in vitro and in vivo against plant pathogens as biocontrol agents. Similarly, microorganisms previously reported as plant growth regulators should be explored as a potential reservoir for isolating microorganisms that could be “transplanted” to other crops. In accordance with this idea, it has been demonstrated that agave endophytes may be transmitted by seeds, providing seedlings with biotic and abiotic stress tolerance and nutrient acquisition [10]. Even most of the microorganisms we found have been previously reported as plant endophytes (Supplementary File S1). Still, some genera are not, suggesting the presence of an *Agave*-specific core yet to be explored as a potential biotechnological reservoir for drought tolerance in other plant species.

The functional characterization of *Agave* endophytes allows us to determine the potential metabolic capabilities of microorganisms present in agave stems and leaves. In congruence with taxonomic clustering, functional clustering effectively separated samples by tissue, except for the leaf sample from *A. karwinskii*, which was grouped with stem samples. The COG category that contributed most to the differences between clusters was energy production and conversion (C), where functions such as carbohydrate catabolism were significantly more abundant in the stem group, suggesting that plants may be filtering microbial communities based on their functional properties and physiological role or compatibility. In *Agave* plants, carbohydrate depolymerization and mobilization during the transition from vegetative to reproductive growth involve a significant physiological shift, enabling the production of the largest flower and one of the highest reproductive efforts known in plants. This catabolic process could be facilitated by microbial endophytic communities that may contribute by producing specialized enzymes. Moreover, the breakdown of agavins and other carbohydrates must rely on specialized microorganisms acting under natural conditions, where spontaneous decomposition occurs. These facts, viewed in light of our results, are very relevant to the *Agave* plant-derived alcoholic beverage industry, as many microbial functions are related to carbohydrate metabolism, particularly those involved in it. In the industry, naturally decaying agave plants are often used to prepare special Tequila batches known as “*Tequila de agave sangrante*” (bleeding-agave Tequila) [14]. The empirical knowledge of incorporating endophytes into factories and fermenting consortia via cross-contamination is critical for preparing these extraordinary batches. Having this in mind, the bioprospection of agave endophytes as a reservoir of first-acting fermenting consortia capable of degrading complex and specialized fructans into less complex carbohydrates that ethanogenic yeasts like *Saccharomyces* can ferment remains one of the most promising fields in the industry. Further exploration of the physiological roles of specific microbial metabolic pathways, selectively overrepresented in each tissue, would reveal insights into plant–microbe interactions at the endospheric level. Most ecological functions rely on physiological responses and metabolic routes, often triggered or mediated by microorganisms. Those predicted functions related to amino acid metabolism, secondary metabolism, and signal transduction also represent a promising field for bioprospecting. The identification of enzymes involved in reactive oxygen species (ROS), phytoalexins, phytohormones, volatile organic compounds (VOCs), toxicants, antibiotics, peptides, and plant physiological responses to drought, chemoreception, or resistance to pathogens in *Agave* endophytes remains completely unexplored.

## 5. Conclusions

Agave endophyte communities comprise a complex mix of microbes, predominantly dominated by bacterial representatives, followed by fungi and a minor proportion of representatives from other kingdoms. These endophytic communities, in contrast to epiphytic communities, appear to be strictly filtered by tissue, regardless of the plant’s degree of domestication or taxonomic position. We found that an essential group of dominant endophytic bacterial genera in agaves has been frequently reported in the fermentation communities of cooked agave juices used for spirits production. The same genera have also been found in the gut of the boring weevil *Scyphophorus acupunctatus*, suggesting microbial horizontal transmission from plants to degradation events. The functional abundance of COGs related to carbohydrate catabolism and energy acquisition in endophytes from stem organs may indicate a potential role of microbial endophytes in the physiology of complex biopolymers and saps of *Agave* plants.

## Figures and Tables

**Table 1:** Species and datasets: Bioproject accession number.

*Agave* Species	Leaves	Stem
*A. americana*	PRJNA998363	PRJNA1190398
*A. angustifolia*	PRJNA956100	PRJNA1190398
*A. cupreata*	PRJNA956101	n/a
*A. fourcroydes*	PRJNA956099	PRJNA1190398
*A. karwinskii*	PRJNA956098	PRJNA1190398
*A. potatorum*	PRJNA956096	PRJNA1190398
*A. rhodacantha*	n/a	PRJNA1190398
*A. tequilana*	PRJNA1010103	n/a

## Data Availability

The original contributions presented in this study are included in the article and Supplementary Materials. Further inquiries can be directed to the corresponding author. The raw sequence dataset is available via the NCBI accession numbers listed in Table 1. The datasets generated during the current study are available from the corresponding author upon reasonable request.

## Supplementary Materials

The following supporting information can be downloaded at: https://www.mdpi.com/article/10.3390/microorganisms14020476/s1, Supplementary File S1: References to reported functional or ecological roles of more abundant taxa.
