# Supplementary material for: Taxonomic and Functional Diversity of Leaves and Stem Endophytes of Eight Agave Species
Source: Microorganisms. 2026 Feb 15;14(2):476. doi: 10.3390/microorganisms14020476 (PMC12943176; doi:10.3390/microorganisms14020476)
Supplement: Supplementary file 1 [file microorganisms-14-00476-s001.zip › microorganisms-4127799-supplementary.pdf]

References to reported functional or ecological roles of more abundant taxa

Genus	Endophyte reference	Fermentation reference	Weevil gut reference
Acinetobacter	https://doi.org/10.1007/s13199-011-0139-x	https://doi.org/10.1007/s11274-006-9175-8	https://doi.org/10.1128/spectrum.02272-22
Actinomadura	https://doi.org/10.1099/ijsem.0.002714		
Aeromonas	https://doi.org/10.3354/ab00422		
Bacillus	https://doi.org/10.1007/s11274-018-2479-7	https://doi.org/10.1016/S0168-1605(01)00706-1	https://doi.org/10.3390/microbiolres15030092
Bradyrhizobium	https://doi.org/10.1128/AEM.02884-20		
Corynebacterium	https://doi.org/10.1007/s00203-022-03101-7		
Enterococcus	https://doi.org/10.1038/s42003-024-05816-3	https://doi.org/10.1016/j.micres.2024.127702	https://doi.org/10.1603/AN13145
Escherichia	https://doi.org/10.1094/PHYTO-08-12-0209-FI	https://doi.org/10.1111/j.1574-6968.1989.tb03398.x	
Frankia	https://doi.org/10.1007/s13199-010-0086-y		
Klebsiella	https://doi.org/10.1007/s11274-025-04300-2	https://doi.org/10.1038/s41598-023-37821-7	https://doi.org/10.1134/S106235902360352X
Leptosphaeria	https://doi.org/10.1007/s44154-024-00186-6		
Leuconostoc	https://doi.org/10.1590/S1517-83822014000400025	https://doi.org/10.1007/978-3-319-23177-8_5	https://doi.org/10.21608/ajs.2018.28385
Ligilactobacillus	https://doi.org/10.1186/s12870-024-04910-2	https://doi.org/10.1007/s00239-024-10189-6	
Limosilactobacillus	https://doi.org/10.3390/fermentation9030228	https://doi.org/10.1016/j.jenvman.2024.121684	
Macrophomina	https://doi.org/10.1007/s12223-022-00976-3		
Penicillium	https://doi.org/10.1007/s13205-020-2081-1	https://doi.org/10.2174/97816080522331110101	
Pseudomonas	https://doi.org/10.3389/fmicb.2015.00745		https://doi.org/10.1186/s12866-019-1690-5
Rhizophagus	https://doi.org/10.1016/j.scienta.2023.112774		
Robertmurraya	https://doi.org/10.1002/agj2.21241		
Saccharomycodes	http://dx.doi.org/10.5772/64806	https://doi.org/10.3390/fermentation4030071	
Salmonella			https://doi.org/10.1111/j.1365-2672.2012.05265.x
Solirubrobacter	https://doi.org/10.1099/ijs.0.057554-0	https://doi.org/10.1016/j.fochx.2023.100618	
Staphylococcus	https://doi.org/10.1007/s00203-020-01911-1		
Streptomyces	https://doi.org/10.1128/AEM.01053-20		
